# Probing the subcellular nanostructure of engineered human cardiomyocytes in 3D tissue

**DOI:** 10.1038/s41378-020-00234-x

**Published:** 2021-01-27

**Authors:** Josh Javor, Jourdan K. Ewoldt, Paige E. Cloonan, Anant Chopra, Rebeccah J. Luu, Guillaume Freychet, Mikhail Zhernenkov, Karl Ludwig, Jonathan G. Seidman, Christine E. Seidman, Christopher S. Chen, David J. Bishop

**Affiliations:** 1 Department of Mechanical Engineering, Boston University, Boston, MA 02215 USA; 2 Department of Biomedical Engineering, Boston University, Boston, MA 02215 USA; 3 SMI Beamline 12-ID, Brookhaven National Laboratory, Upton, NY 11973 USA; 4 Department of Physics, Boston University, Boston, MA 02215 USA; 5 Division of Materials Science, Boston University, Boston, Massachusetts 02215 USA; 6 Department of Genetics, Harvard Medical School, Boston, MA 02215 USA; 7 Department of Electrical Engineering, Boston University, Boston, MA 02215 USA

**Keywords:** Nanostructures, Nanophotonics and plasmonics, Organic-inorganic nanostructures

## Abstract

The structural and functional maturation of human induced pluripotent stem cell-derived cardiomyocytes (hiPSC-CMs) is essential for pharmaceutical testing, disease modeling, and ultimately therapeutic use. Multicellular 3D-tissue platforms have improved the functional maturation of hiPSC-CMs, but probing cardiac contractile properties in a 3D environment remains challenging, especially at depth and in live tissues. Using small-angle X-ray scattering (SAXS) imaging, we show that hiPSC-CMs matured and examined in a 3D environment exhibit a periodic spatial arrangement of the myofilament lattice, which has not been previously detected in hiPSC-CMs. The contractile force is found to correlate with both the scattering intensity (*R*^2^ = 0.44) and lattice spacing (*R*^2^ = 0.46). The scattering intensity also correlates with lattice spacing (*R*^2^ = 0.81), suggestive of lower noise in our structural measurement than in the functional measurement. Notably, we observed decreased myofilament ordering in tissues with a myofilament mutation known to lead to hypertrophic cardiomyopathy (HCM). Our results highlight the progress of human cardiac tissue engineering and enable unprecedented study of structural maturation in hiPSC-CMs.

## Introduction

The engineering of 3D human cardiac tissue has advanced significantly in the last decade, promoting the multicellular organization and functional maturation of human induced pluripotent stem cell-derived cardiomyocytes (hiPSC-CMs)^1–3^. While this is promising for applications such as drug development^4^, disease modeling^5,6^, and therapeutic implants^7^, hiPSC-CMs require further maturation and improved reproducibility^8,9^. These needs have inspired many new technologies, such as three-dimensional (3D) tissue platforms^1–3^, active mechanical environments^10^, tissue training protocols^1,2^, and techniques to probe subcellular information within the 3D environment^11–15^, all of which have informed new maturation protocols for cardiac tissue. Despite the critical importance of subcellular structure to cardiac tissue function and maturation, few tools exist to probe the nanostructural organization of hiPSC-CMs in a 3D environment, especially in live tissue.

In the engineering of tissues from hiPSC-CMs, complex 3D organization and accessibility to subcellular information are areas of significant research activity. In the in vivo heart, complex 3D organization is instrumental to cardiac function. In vitro models with an engineered 3D environment promote improvements in structure and function relative to their two-dimensional (2D) monolayer counterparts^16–18^. Specifically, 3D models have been shown to improve cell morphology, contraction ability, the presence of intracellular adhesion structures, organization of myofibrils, mitochondria morphology, endoplasmic reticulum contents, and the expression of cardiac differentiation markers^16^. In parallel, subcellular techniques have been developed to probe function, with readouts such as action potential^11,12^, traction force^13^, and metabolism^14^, but most of these techniques are limited to 2D and surface readouts.

**Fig. 1:**
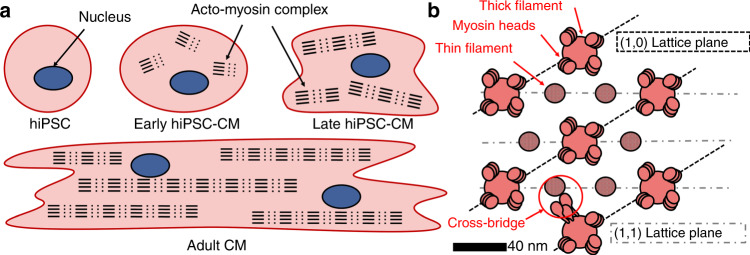
Actomyosin model in cardiac tissue. **a** Structural maturation of sarcomere in hiPSC-CMs (adopted from^22^). **b** Ideal cross-section of an actomyosin complex within the sarcomere of a mature CM (as in adult cardiac muscle). For scale, the shortest spacing between actin filaments is typically 37–45 nm in vertebrate cardiac muscle^4,5^

Advances in 3D tissue culture require measurement techniques that can probe depth and live dynamics. Electron microscopy requires invasive sample preparation, chemical fixation, microtome slicing, staining, and a high vacuum environment^1,2,16^. Not only do these invasive methods have the potential to distort structural features, but the throughput is low, and this technique is not compatible with live tissue imaging. Light-based methods are typically compatible with live tissue, but fundamentally face the diffraction limit and cannot be used to directly view nanometer-scale structures, such as the filaments and myosin heads within the sarcomere. Fluorescent and confocal microscopy have achieved the speeds and resolutions required for such analysis, but not at depth; submicron imaging techniques have largely stalled at <100 µm from the surface^19,20^. This has led to the recent development of creative techniques such as whispering gallery mode microlasing^15^, but this still requires the invasive sample preparation of inserting a microbead; moreover, it only measures structures within 200 nm of the bead, and the signal-to-background ratio is near 3 dB at depths greater than 200 µm. As such, the present understanding of structural maturation in hiPSC-CMs (Fig. 1a)^8,18^ has been largely developed from 2D models and the aforementioned techniques, and information at depth or in live tissue is difficult to achieve. Alternative approaches to probe subcellular structure in 3D models will provide critical information, tying structure to function, in the modeling and maturation of hiPSC-CMs.

Small-angle X-ray scattering (SAXS) has been established as a powerful tool for probing nanoscale structures in highly ordered materials and is capable of measuring live tissue dynamics at depth in adult cardiac muscle^21,22^ (Fig. 1a, bottom). In myofilaments, which are the primary contractile units within cardiomyocytes, actomyosin complexes form a hexagonal lattice of filaments that can be detected by SAXS (Fig. 1b). The scattering intensities of distinct planes relative to myosin and actin provide a direct measure of myofilament lattice order and spacing, which are modulated by the contractile state of the muscle^21–27^. The (1,0) plane can be defined to contain “thick” myofilaments, while the (1,1) plane consists mainly of “thin” actin filaments. During contraction, myosin heads extend off the myofilament to form cross-bridges, and the mass is transferred from the (1,0) plane to the (1,1) plane. The ratio of the scattering intensities of these two planes is analyzed, and the (1,0) intensity is typically much larger than the (1,1) intensity in adult cardiac muscle^21–27^. Despite advances in cardiac SAXS techniques and functional maturation in engineered tissue platforms, hiPSC-CMs have not yet been shown to have a nanoscale order detectable by SAXS^27^. Notably, tissues grown in these 3D platforms still exhibit at least a tenfold lower contractile force than adult cardiac muscle. Given that alignment and striation at the cellular scale have been shown to improve contractile force^1,2^, this shortfall in functional maturation may be due to misalignment of the subcellular structure, which largely comprises the myofilament lattice. As such, we refer to the improvement in myofilament lattice ordering as structural maturation.

Regardless of tissue scale or dimension (2D and 3D), electrophysiology^28^ and contractile force (CF) generation^1–3^ are the primary metrics of functional maturity in engineered heart tissues. The ability to assess the nanoscale structure of engineered tissues would add an important dimension to the characterization of structural maturation. Here, we leverage the periodic arrangement of myofilaments in mature hiPSC-CMs to detect structural maturation with SAXS. We develop a robust batch-process methodology to conduct measurements on a high-throughput 3D tissue platform. Both myofilament scattering intensity and lattice spacing are shown to correlate with contractile force in hiPSC-CMs. We show that these new techniques are capable of detecting significant structural differences in hiPSC-CMs arising from a myofilament mutation in a genetic model of hypertrophic cardiomyopathy (HCM) compared to a wild-type control. This work highlights the utility of 3D tissue platforms with an engineered mechanical environment and the necessity for methodologies to probe maturation in such advanced 3D tissues.

## Results and discussion

**Fig. 2:**
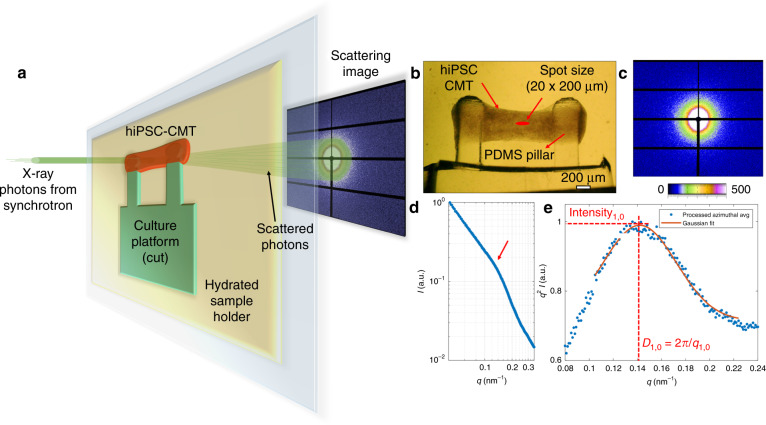
SAXS in hiPSC-CMs. **a** Schematic of synchrotron X-ray scattering of 3D tissue on passive mechanical platform. **b** Platform with hiPSC-CMs grown in a 3D microtissue environment. **c** Typical SAXS image from the center of a tissue, where the color bar is units of intensity (photons). **d** Azimuthal average of scattering image in **c**. **e** *q*^2^ weighted peak in **d** fit by a Gaussian

The primary objectives of this study were to develop a robust methodology for probing the nanostructure of hiPSC-CMs in a 3D environment, to elucidate the structures contributing to the signal, to validate identified trends in SAXS results from explanted cardiac tissue and to apply the newly developed technique to a genetic model in hiPSC-CMs. For this to be successful, a platform with the ability to detect both functional and structural properties at a moderately high throughput is necessary to generate statistics. Therefore, the microtissue strain gauge (µTUG) platform^10,29^, where batches of cardiac microtissues (CMTs) are examined, was selected. The hiPSC-CMs were cocultured with human mesenchymal stem cells and suspended in a 3D fibrin matrix (see Methods). The elastomer pillars are designed with a spherical top to facilitate and maintain tissue attachment. The resulting 3D tissue (~1.2 mm long, 500 µm diameter) compacts for several days and contracts spontaneously in the passive mechanical environment (Fig. 2b). Deflections of the elastomer pillars are tracked by a microscope and provide a functional readout of peak tissue contractile force. All presented samples exhibited contractile forces within the reported hiPSC-CMT range^1,2^.

The contractile apparatus of hiPSC-CMs was expected to be loosely organized during early stages of cardiac maturation (illustrated in Fig. 1a)^8,9^. Therefore, to detect the structure, samples were probed by synchrotron SAXS, where a high-intensity beam of X-ray photons is transmitted through the sample and scatters elastically off of nanoscale structures at small angles, producing a scattering image recorded with a 2D detector (Fig. 2a). In this work, the measurements were performed at the SMI beamline NSLS-II at Brookhaven National Laboratory. It is noteworthy that scattering experiments have been conducted previously on a similar tissue system, but the level of hiPSC-CM maturation was determined to be insufficient to discern a signal from the underlying structure^27^. In our experiment, tissue function was recorded first in live tissue, and then SAXS was conducted on the same samples fixed at room temperature. During the SAXS measurement, CMTs remained on the posts and were kept hydrated by a buffer solution (see Methods section). A spot size of 20 μm × 200 μm in the center of the tissue (Fig. 2b) was irradiated, producing a scattering image (Fig. 2c).

To ensure a robust methodology, a customized routine was developed to process the pixel intensities in the scattering images. A circular average of the representative sample (Fig. 2b) after background subtraction is shown (Fig. 2d). A Kratky plot, where the signal is multiplied by the square of the wavenumber (*q*), is commonly used to extract information from a loosely organized sample^30^. Here, Gaussian fit parameters of the intensity profile (Fig. 2e) were extracted and compared in a batch process. In the loosely ordered hiPSC-CMs, the peak intensity is assumed to be largely representative of structural order. The center of the peak in inverse space corresponds to the lattice spacing. The full width at half maximum (FWHM, Δ*q*) is related to the length over which order persists (correlation length) in a sample with homogenous lattice strain.

The anatomical source of scattering was discerned from the wavenumber (*q*), literature discussing cardiac SAXS^21–27^, and a series of experiments with varying matrix and cell conditions. The wavenumber is defined as *q* = (4*π*/*λ*)sin(*θ*), where *θ* is the scattering angle and *λ* is a constant wavelength. The intensity is the number of scattered photons radially integrated over the pixels of the detector. In the representative sample (Fig. 2d), *q* was equal to 0.14 nm^−1^, corresponding to a spacing of *D* = 44 nm. This is in the range of typical spacing found for a myofilament lattice in mature tissue^21,24,26,27^. To verify the attribution of the *q* = 0.14 nm^−1^ peak to the myofilament lattice, we treated tissues with collagenase or trypsin, enzymes that digest components of the extracellular matrix (ECM). If the observed scattering peak were due to ordering in the ECM, chemical distortion, or degradation of the ECM would affect or diminish that peak. However, we found that the peak was preserved in enzyme-treated tissues (Fig. S1). Furthermore, we found that tissues containing only nonmyocytes and decellularized hiPSC-CM tissues did not exhibit a peak near this wavenumber (Fig. S1). This reaffirms the assertion that the peak at *q* = 0.14 nm^−1^ is representative of the myofilament lattice.

Further information may be extracted from the data regions outside of the peak at *q* = 0.14 nm^−1^. As mentioned earlier, the structural order is assumed to be loosely organized^30^. To assess the structural geometry of a loosely organized lattice, the sample was assumed to comprise elongated nanoscale structures^31^. Form factors were then extracted from the Porod plot (Fig. 2d) in the low and intermediate Guinier regions (Fig. S2) to find an average cylindrical geometry of 22 nm radius and 60 nm length. Previous work using electron microscopy has found diameters of individual myofilament diameters of 30–40 nm and actin filaments of 10 nm^32^. This average geometry over the scattering volume may be suggestive of fragmented fibers or segmented contractile apparatuses in maturing hiPSC-CMs (as illustrated in Fig. 1a)^8^.

An analysis of the myofilament lattice in hiPSC-CMs compared to adult cardiac tissue provides a roadmap for structural maturation. Since the X-ray beam passes through the entire tissue, this technique reports a volumetric average with contributions from multiple cells. The myofilament lattice spacing, *D*_1,0_, of a representative sample (Fig. 2b) was found to be 44 nm, and the FWHM was Δ*q*_1,0_ = 0.07 nm^−1^, for a correlation length of ~100 nm. Compared to adult cardiac muscle, where *D*_1,0_ has been reported to be near 39 nm (refs. ^23–25^), hiPSC-CMs exhibited a slightly larger *D*_1,0_ and a shorter correlation length. The assertion that contractile units are ordered over a smaller region compared to adult tissue may provide a structural perspective for the reduced contractile function seen in hiPSC-CMs.

**Fig. 3:**
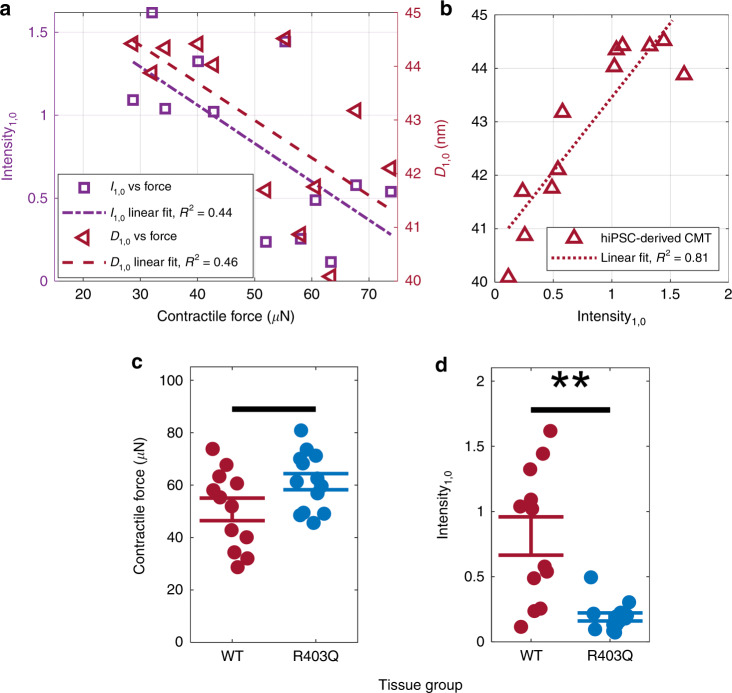
Scattering results in hiPSC-CMs. **a** Negative correlation between CF and *I*_1,0_ as well as *D*_1,0_ (pCa 2.7). **b** Positive correlation between both SAXS parameters in **a**. **c** CF of tissue groups, WT and R403Q^+/−^ (*p* = 0.058). **d** I_1,0_ of tissue groups, where the trend varies inversely to CF in **c**, suggesting more cross-bridge formation in R403Q^+/−^ hiPSC-CMs

With both structural and functional information from the same hiPSC-CMs, we were able to directly compare the SAXS information with the contractile force. To accomplish this, we simply calculated the correlation coefficient between the structural and functional datasets. Here, the buffer contained a calcium concentration of pCa 2.7. *D*_1,0_ had a negative relationship with CF (*R*^2^ = 0.46), suggesting that a tissue capable of stronger CF has a more compact lattice (Fig. 3a). A negative relationship with (1,0) scattering intensity, *I*_1,0_, (*R*^2^ = 0.44) suggests that more cross-bridges were formed in a tissue with a higher CF. Furthermore, there was a stronger correlation between *I*_1,0_ and *D*_1,0_ (*R*^2^ = 0.81), suggesting greater variability in CF measurements than in *I*_1,0_ and *D*_1,0_ (Fig. 3b). This difference may be because SAXS sampled from only a spot in the center of the CMT, whereas the CF was measured in an entire CMT.

To understand how disease-associated myofilament mutations might impact lattice organization, we compared a cell line with the HCM-causing myofilament mutation R403Q^+/−^ with an isogenic wild-type (WT) control hiPSC line. The CF of R403Q^+/−^ CMTs was slightly higher than that of WT CMTs (*p* = 0.058; Fig. 3c). The CF of a CMT is dependent on the number of hiPSC-CMs and nonmyocytes in the tissue, creating variability, but significantly higher forces have been observed in single hiPSC-CMs of the same cell lines previously^5^. Conversely, the mean *I*_1,0_ for R403Q^+/−^ hiPSC-CMs was found to be significantly lower than that for the WT control (Fig. 3d). This suggests that more cross-bridges were formed in R403Q^+/−^ hiPSC-CMs than in WT CMs, consistent with prior work in adult cardiac muscle^24^.

**Fig. 4:**
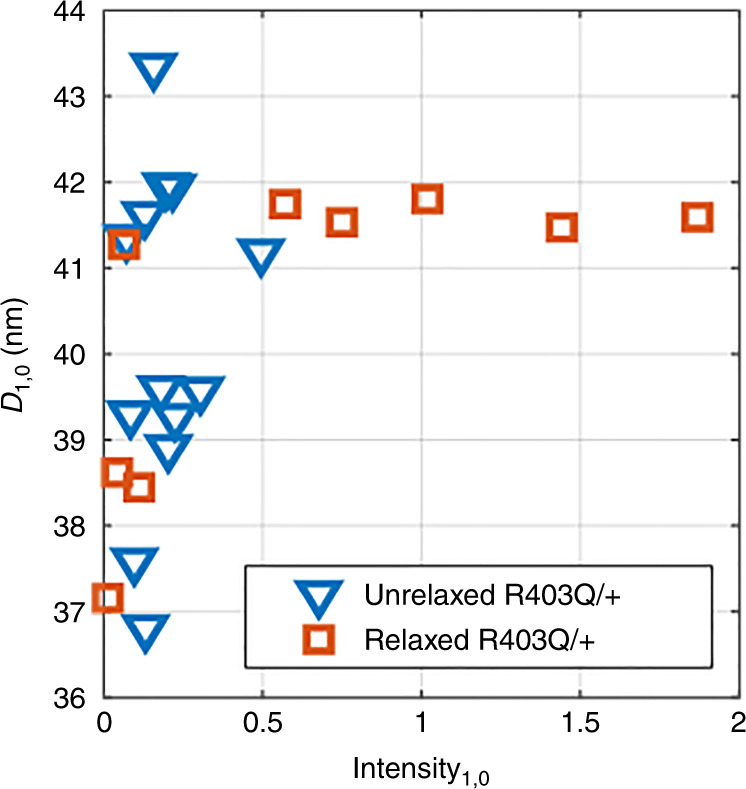
Effect of relaxation buffer in hiPSC-CMs. The effect of a 50 mM K+ relaxation buffer on tissues with the myofilament mutation, producing higher intensity and more consistent *D*_1,0_

We also analyzed the effect of a relaxation buffer on the contractile structure (Fig. 4). Relaxation is known to cause myosin heads to aggregate toward the myofilament in adult cardiac muscle^21–27^, so it is expected that relaxation of the contractile apparatus will increase the scattering intensity of the myofilament lattice at (1,0). Hypercontractility in tissues with the R403Q^+/−^ mutation may be explained by a higher activity of the myosin heads, extending away from the myofilament, and forming cross-bridges with the actin filament^33^. Therefore, we chose to examine the effect of a relaxation buffer on R403Q^+/−^ CMTs. We observed that *I*_1,0_ increased in several relaxed samples, consistent with our hypothesis. Furthermore, the distribution of *D*_1,0_ for those higher intensity samples (*I*_1,0_ > 0.5) narrowed significantly, suggesting an improved ordering of the nanoscale structure when in a relaxed state.

**Fig. 5:**
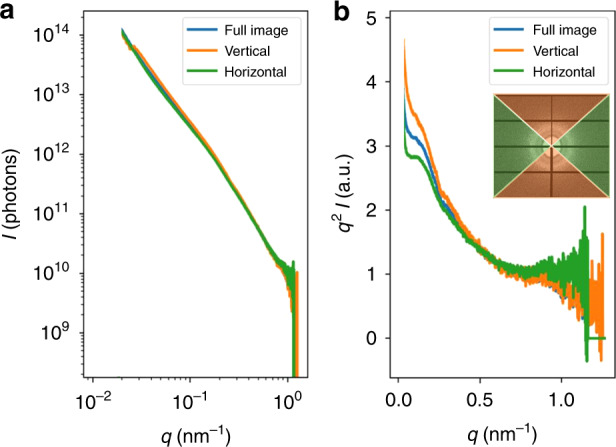
Anisotropic structure in hiPSC-CMs grown in 3D CMTs. Circular averages of the scattering image in Fig. 2 showing the Porod plot (**a**) and enhanced peaks in the Kratky plot (**b**). Vertical and horizontal show the circular average of only the regions corresponding to that color in the inset

It is noteworthy that while the scattering has been observed to be largely isotropic (Fig. 2c), a small degree of anisotropy can be detected (Fig. 5). The circular average (left) and *q*^2^-weighted intensity before normalization (right) are shown. The scattering image is parsed into scattering in the vertical (perpendicular to tissue in Fig. 2b) and horizontal (along tissue in Fig. 2b) directions. Filaments oriented in the horizontal direction will scatter vertically, and those oriented in the vertical direction will scatter horizontally. Tissues predominantly scatter in the vertical direction, indicating that the myofilament lattice is primarily ordered along the tissue, which allows it to produce net contraction along a single axis, deflecting the pillars.

**Fig. 6:**
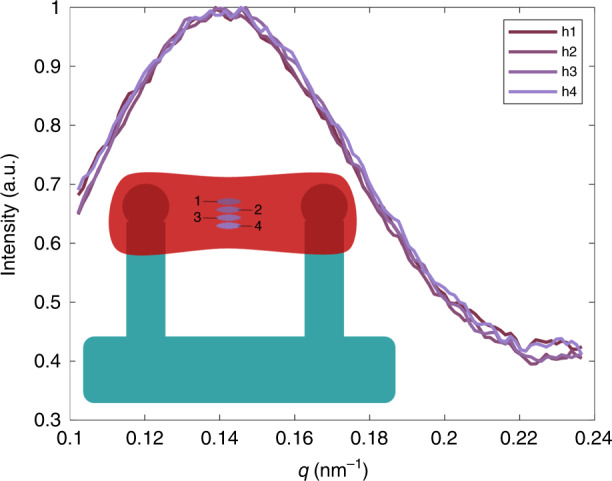
Effect of scattering location. Within a 3D cardiac tissue, cell density, tissue thickness, and cardiac nanostructure may vary. The Porod invariant (area under peak) is consistent after a concentration (volume) correction in the batch-process. The peak shape is consistent at different spots in the sample, suggesting that nanostructural order is similar within a given sample

A 3D tissue environment may be heterogeneous, and the thickness of the sample, cardiomyocyte density, and cardiomyocyte nanostructure vary with position. The results presented in Figs. 2–5 are gathered from a single spot in the center of cardiac tissue where the thickness and cardiomyocyte density are assumed to be maximal. To understand the heterogeneity (and thickness dependence) within cardiac tissue, we varied the scattering location, as shown in Fig. 6. Here, the batch postprocessing is used as for other data. Then, the dataset is divided by the average of the low-*q* region (here defined as 0.07 < *q* < 0.10), effectively normalizing the data. Since the intensity is scaled by the low-*q* region during batch-processing analysis, the tissue is effectively normalized. This is a common scattering technique for hydrated biological samples with low signal-to-noise ratio^30^. The area under the curve of the peak is called the Porod invariant, which is independent of sample concentration and volume. After scaling, the shape of the peak is expected to be equivalent for scattering measured at different positions in the tissue. The scattering peaks in Fig. 6 have consistent overlap, suggesting that the structure is relatively consistent in a given tissue sample. Additionally, the nanostructural order is far more similar within a given tissue sample than among different samples, as is reported in Figs. 3 and 4, where both peak amplitude *I*_1,0_ and peak width *D*_1,0_ change. While SAXS averages the scattering of all cells in the volume of the scattered tissue (in our case, an approximate volume of 0.002 mm^3^), any changes in cell density do not appear to contribute to large variations within a sample. Therefore, despite the heterogeneous 3D tissue environment, we find that our technique and analytic approach is sufficient for nanostructural analysis of the myofilament lattice in hiPSC-CMs.

We hypothesize that there is a threshold of maturation for myofilament detection, as samples exhibiting contractile forces <20 μN did not show adequate scattering signals (and were therefore excluded). Despite the ability of the hiPSC-CMs to functionally contract the tissue, the actin filament lattice peak (1,1) was not observed in the scattering signal. In typical cardiac SAXS analysis of mature tissue, a ratio of the myofilament to actin filament lattice peak intensities (*I*_1,1_/*I*_1,0_) is reported, where the (1,0) signal is much stronger. Therefore, the (1,1) signal may be obscured by noise or by the large width of the (1,0) signal peak.

## Conclusion

Presently, a large part of pharmaceutical research still relies on expensive and inaccurate animal models. Engineered human cardiac tissue not only will provide greater relevance to human studies but has potential for use in patient-specific and heart-on-a-chip trials. These applications require a greater maturity of cardiac cells and have been improved by multicellular 3D tissue platforms. However, methods for live measurement of cardiac dynamics and maturation in such a 3D environment are lacking. Electron microscopy is very useful but requires highly invasive preparation in a vacuum environment. Fluorescent and confocal microscopy have achieved the speeds and resolutions required for analyzing many contractile properties, but these techniques are largely limited to depths <100 µm from the surface of 3D tissue. SAXS has been shown to be a promising alternative method for the nanostructural measurement of live tissue dynamics and contractile properties, and our work is the first step in that direction for engineered cardiac tissue. Here, we demonstrate that engineered cardiac cells grown from hiPSCs in a 3D tissue environment produce internal nanostructures exhibiting periodic spatial arrangement of a high degree. We conclude with the observation of the myofilament lattice from the peak position in inverse space, and we discuss the effects of tissue preparation, structural anisotropy, and sample heterogeneity on scattering results. Compared to adult cardiac muscle, we found that hiPSC-CMs exhibit diminished nanoscale ordering, but follow the expected relationships with contractile force, the HCM-causing R403Q^+/−^ mutation, and relaxation buffer application. While this is an indication of hiPSC-CM maturation in 3D constructs, recent advances in hiPSC-CM maturation using electromechanical stimulation are ideal candidates for further study.

## Materials and methods

For the generation and recording of hiPSC-derived CMTs, hiPSCs from the PGP1 parent line and CRISPR-Cas9 PGP1-edited cells with a heterozygous R403Q mutation in the β-myosin heavy chain (MYH7) were received from the Seidman Lab^5^. The hiPSCs were maintained in mTeSR1 (StemCell) on Matrigel (Fisher) mixed 1:80 in DMEM/F-12 (Fisher) and split using Accutase (Fisher) at 60–90% confluence. iPSCs were differentiated into iPSC-CMs by small-molecule, monolayer-based manipulation of the Wnt signaling pathway^34^. Once cells were beating, iPSC-CMs were purified using RPMI no-glucose media (Fisher) with 4 mM sodium DL lactate solution (Sigma) for 2–5 days. Following selection, cells were replated and maintained in RPMI with 1:50 B-27 Supplement (Fisher) on 10 µg/mL fibronectin (Fisher)-coated plates until day 30+.

CMT devices with tissue wells, each containing two cylindrical micropillars with spherical caps, were cast in PDMS from a 3D-printed mold (Protolabs), similar to a previous design^10^. Devices were plasma treated for 60 s, treated with 0.01% PLL (ScienCell) for 2 h and 0.1% glutaraldehyde (EMS) for 15 min, washed 3x with DI water, and let sit in DI water at 4 °C overnight. Immediately prior to seeding, devices were soaked in 70% ethanol for 30 min, dried, and UV-sterilized for 15 min. Next, 3 µL of 5% BSA was added to the bottom of each tissue well, and the devices were centrifuged at 3000 rpm for 1.5 min. After 1 h of incubation at RT, BSA was removed, and 2 µL of 2% Pluronic F-127 (Sigma) was added to each well and incubated for 30 min at RT to prevent CMTs from adhering to the bottom surface of the devices.

A total of 60,000 cells per CMT, 90% iPSC-CMs and 10% human mesenchymal stem cells (hMSCs), were mixed in 7.5 µL of an ECM solution, 4 mg/mL human fibrinogen (Sigma), 10% Matrigel (Corning), 1.6 mg/mL thrombin (Sigma), 5 µM Y-27632 (Tocris), and 33 μg/mL aprotinin (Sigma). The cell-ECM mixture was pipetted into each well, and after polymerization for 5 min, growth media containing high-glucose DMEM (Fisher) supplemented with 10% fetal bovine serum (Sigma), 1% penicillin–streptomycin (Fisher), 1% nonessential amino acids (Fisher), 1% GlutaMAX (Fisher), 5 µM Y-27632, and 33 μg/mL aprotinin was added and replaced every other day. Y-27632 was removed 2 days after seeding, and aprotinin was decreased to 16 μg/mL after 7 days. Time-lapse videos of CMT contraction were then acquired at 30 fps using a ×4 objective on a Nikon Eclipse Ti with an Evolve EMCCD camera (Photometrics) equipped with a controlled environment chamber. CF measurements were calculated from pillar deflection and the measured stiffness of the pillar (2.67 N/m)^29^. After recording, CMTs were fixed in 4% PFA (Fisher) for 30 min, washed 3x with PBS, and stored in PBS until SAXS radiation. CMTs fixed in relaxation buffer were placed in growth media with 50 mM KCl for 1 h and fixed in 4% PFA in growth media with 50 mM KCl for 30 min.

For SAXS and associated data processing, tissues were placed between 70-μm-thick glass and Kapton tape, remaining stretched on the pillars and in a PBS solution at room temperature. The X-ray wavelength was 0.077 nm, the sample-to-detector distance was 8 m, the flux was 1 × 10^12^ ph/s, and the samples were irradiated for 1.5 s at a 20 × 200 μm^2^ spot. This results in a calculated radiation dosage of 0.15 MGy, which is lower by a factor of 20 than the dose used in previous work with hydrated cardiac cells^27,35^ (further detail in Supplementary Material). SAXS images were recorded by a Pilatus3 X 1M detector and batch-processed in a Python-based Jupyter notebook using a combination of data processing techniques, as follows. First, the scattering from the tissue is circularly averaged to extract an intensity versus wavenumber profile. Background data are collected for each sample by selecting a spot where there is only buffer in the chamber (no tissue) and subtracting from experimental curves. Slope subtraction is employed by fitting the intensity decay in regions outside of our peak of interest and subtracting the fit. Finally, the region of interest is fit by a Gaussian peak, where all the postprocessing settings are consistent for all samples in a batch process. Student’s *t* test was used to determine significant differences between CMT groups (**p < 0.005). Error bars show the standard error of the mean.

## Supplementary information

Supplementary Information

Data Set 1
