# Supplementary material for: Probing the subcellular nanostructure of engineered human cardiomyocytes in 3D tissue
Source: Microsyst Nanoeng. 2021 Jan 27;7:10. doi: 10.1038/s41378-020-00234-x (PMC8433147; doi:10.1038/s41378-020-00234-x)
Supplement: Supplementary file 1 — Supplementary Information [file 41378_2020_234_MOESM1_ESM.docx]

Supplementary Information

Source of detected SAXS signal

|  | **PFA Concentration** |  | **Cells during culture** | **Cells during SAXS** | **Treatment** |
| --- | --- | --- | --- | --- | --- |
| T1 | 4% |  | hMSCs + hiPSC-CMs | hMSCs + hiPSC-CMs | - |
| T2 | 4% |  | hMSCs | hMSCs | - |
| T3 | 8% |  | hMSCs | hMSCs | - |
| T4 | 4% |  | hMSCs + hiPSC-CMs | hMSCs + hiPSC-CMs | Collagenase |
| T5 | 4% |  | hMSCs + hiPSC-CMs | hMSCs + hiPSC-CMs | Trypsin |
| T6 | 4% |  | hMSCs + hiPSC-CMs | decellularized | - |
| T7 | 4% |  | hMSCs + R403Q^+/-^ hiPSC-CMs | decellularized | - |

**Supplementary Table 1**: Variations in microtissues before SAXS. Note that only T1, T4 and T5 contain hiPSC-CMs at the time of SAXS.

**Supplementary Figure 1**: Kratky plots of CMTs in Table S1. Note that only tissues containing hiPSC-CMs exhibit a peak near q=0.14nm^-1^. Plots are not normalized, as the presence of certain features is compared only.

In order to determine the source of the peak at q=0.14nm^-1^, as seen in 3D multicellular tissues containing hiPSC-derived cardiomyocytes (Figure 2e), several conditions are varied before conducting SAXS (Figure S1 and Table S1). This is a complex problem because each variable could alter the final nanostructure after culture or maturation (if containing cardiomyocytes). All tissues are cultured and post-processed by the same procedure (see Methods), with variations listed (Table S1). Tissue 1 (or T1), is the representative sample shown earlier (Figure 2e). Sample T2 contains only human mesenchymal stem cells (hMSCs), and therefore does not contract without CMs. T3 is similar to T2, but is fixed with double the concentration of paraformaldehyde (PFA) as compared to T2. Fixation with PFA has been shown to minimize distortion of intracellular structures, but may affect the extracellular matrix^1^. T4 and T5 are similar to T1, but are treated before fixation with collagenase and trypsin, respectfully. These two enzymes are known to selectively degrade the extracellular matrix by severing peptide bonds^2^, which is expected to distort the extracellular matrix. Additionally, T4 and T5 exhibited very low contraction force (less than 20µN), which screened them from typical analysis. Sample T6 is similar to T1, but is decellularized with an agent that selectively degrades the cells and leaves the matrix behind. T7 is similar to T6, where the hiPSC-CMs are R403Q^+/-^ gene-edited cells. The holes left behind in the matrix in T6 and T7 may cause scattering at the boundary interfaces, distorting the scattering profile^3^.

The scattering profiles of each tissue from Table S1 is shown in Figure S1. We do not normalize or fit this data with the methods developed for hiPSC-CMTs (Materials and Methods) because the qualitative analysis here is based on the presence of certain features, such as the peak at q=0.14nm^-1^. It is noteworthy that the only tissues containing hiPSC-CMs at the time of SAXS (T1, T4 and T5) contain a peak near q=0.14nm^-1^. We also find that peak was preserved in enzyme treated tissues (Figure S1). If the observed scattering peak at q=0.14nm^-1^ were due to ordering in the ECM, chemical distortion or degradation of the ECM in T4 and T5 would affect or diminish that peak. Furthermore, we find that tissues with only non-myocytes as well as decellularized hiPSC-CM tissues do not exhibit a peak near q=0.14nm^-1^. These are the only tissues that could contain the contractile apparatuses of a late-maturation-stage hiPSC-CM depicted earlier (Figure 1a, top right). Moreover, the peak position, q=0.14nm^-1^, is in the range of previously reported scattering from the myofilament lattice^4-10^. The myofilament peak also exhibits the highest intensity relative to other structures in cardiac tissue^4-10^, and so it would be the first peak expected to be seen in a loosely ordered sample.

Geometry estimation from q-range

**Supplementary Figure 2**: Guinier regions viewed in a Porod plot, where fits are extracted to examine structural geometry.

A technique to estimate geometry in a sample lattice has been outlined in detail earlier^11^. For elongated objects (such as a cylinder), the Guinier-Porod plot (Figure 2d and S2) can be broken up into three regions: low-q (q<0.09), intermediate-q (0.09<q<0.9), and high-q (q>0.9). The range of collected data in this work encompasses the low-q and intermediate-q regions. In order to selectively fit those regions (shown by yellow and orange in Figure S2), the peak from the hiPSC-CMs must be excluded (shown by blue markers in Figure S2). The low-q range is fit by Eq 1 and the intermediate-q range is fit by Eq 2, below. From fits of the low-q and intermediate-q data, the *R­­­_g_* variables are extracted. These are then used in Eq 3 and Eq 4 to calculate *L* and *R*. Here, *R_g_* is the radius of gyration, *R* is the cylinder radius, and *L* is the cylinder length. In the case of our representative sample, the radius and length of the cylinder is found to be approximately 22nm and 60nm, respectfully.

$I\left( q \right)=I_{0}e^{-\frac{q^{2}R_{g,low}^{2}}{3}}$ (low-q) Eq (1)

$I\left( q \right)=\frac{I_{0}}{q}e^{-\frac{q^{2}R_{g,int}^{2}}{2}}$ (intermediate-q) Eq (2)

$R_{g,low}^{2}=\frac{R^{2}}{2}$ Eq (3)

$R_{g,int}^{2}=\frac{L^{2}}{12}+\frac{R^{2}}{2}$ Eq (4)

Consideration of radiation damage

Damage to soft matter such as cardiac cells and tissue is typically calculated by the maximum allowable dosage^10,12,13^. This is calculated by the Equation 5 below.

$D=\frac{\mu}{\rho}\frac{I_{0}\tau E}{\Delta x\Delta y}$ Eq (5)

Here, *D* is the dosage due to radiation (Gy, or J/kg). The ratio μ/ρ is the mass attenuation coefficient, and is calculated by linear interpolation from the XAAMDI database for muscle tissue to be 1.501cm^2^/g [12]. I_0_ is the radiation intensity (1x10^12 ph/s). τ is the exposure time (1.5s). E is the energy (16.1keV). And ∆x∆y is the spot size (20x200µm). As a result, the dosage of a typical experiment in our case is 0.15 MGy. This is over a factor of 20 lower than previous studies with hydrated cardiac cells, where a dosage of 3.6 MGy is used [10,13]. This suggests that the SAXS measurement parameters used in this work were not damaging the tissue structures.

REFERENCES

1. Li, Yue, et al. "The effects of chemical fixation on the cellular nanostructure." Experimental cell research 358.2 (2017): 253-259.
2. Krane, Stephen M. "Collagenases and collagen degradation." Journal of Investigative Dermatology 79.1 (1982): 83-86.
3. Schmidt, Paul W., et al. "Small‐angle x‐ray scattering from the surfaces of reversed‐phase silicas: Power‐law scattering exponents of magnitudes greater than four." The Journal of chemical physics 94.2 (1991): 1474-1479.
4. Ait-Mou, Younss, et al. "Titin strain contributes to the Frank–Starling law of the heart by structural rearrangements of both thin-and thick-filament proteins." PNAS 113.8 (2016): 2306-2311.
5. Brunello, Elisabetta, et al. "Myosin filament-based regulation of the dynamics of contraction in heart muscle." PNAS 117.14 (2020): 8177-8186.
6. Yuan, Chen-Ching, et al. "Sarcomeric perturbations of myosin motors lead to dilated cardiomyopathy in genetically modified MYL2 mice." PNAS 115.10 (2018): E2338-E2347.
7. Colson, Brett A., et al. "Differential roles of regulatory light chain and myosin binding protein-C phosphorylations in the modulation of cardiac force development." J. of Physiology 588.6 (2010): 981-993.
8. Anderson, Robert L., et al. "Deciphering the super relaxed state of human β-cardiac myosin and the mode of action of mavacamten from myosin molecules to muscle fibers." PNAS 115.35 (2018): E8143-E8152.
9. Martyn, Donald A., et al. "Response of equatorial x-ray reflections and stiffness to altered sarcomere length and myofilament lattice spacing in relaxed skinned cardiac muscle." Biophys. J. 86.2 (2004): 1002-1011.
10. Nicolas, Jan-David, et al. "X-ray diffraction imaging of cardiac cells and tissue." Biophys. and Molecular. Bio. 144 (2019): 151-165.
11. Hammouda, Boualem. "A new Guinier–Porod model." Journal of Applied Crystallography 43.4 (2010): 716-719.
12. Howells M R, Beetz T, Chapman H N, Cui C, Holton J M, Jacobsen C J, Kirz J, Lima E, Marchesini S, Miao H, Sayre D, Shapiro D A, Spence J C H, Starodub D (2009) An assessment of the resolution limitation due to radiation-damage in x-ray diffraction microscopy. J. Electron Spectrosc. Relat. Phenom. 170:4–12
13. Nicolas, Jan-David, et al. "X-ray diffraction imaging of cardiac cells and tissue." Biophys. and Molecular. Bio. 144 (2019): 151-165.
